# Supplementary material for: Resolving the full spectrum of human genome variation using Linked-Reads
Source: Genome Res. 2019 Apr;29(4):635–45. doi: 10.1101/gr.234443.118 (PMC6442396; doi:10.1101/gr.234443.118)
Supplement: Supplemental Material [file supp_gr.234443.118_Supplemental_longranger-2.2.zip › longranger-2.2.tar.bz2!/lr2.2-public/lib/stan/cmdstan/stan/doc/api/html/agrad_2fwd_2prob_2univariate_2continuous_2normal_8hpp_source.html]

Stan: src/stan/agrad/fwd/prob/univariate/continuous/normal.hpp Source File


|  |  |
| --- | --- |
|  | Stan  2.5.0  probability, sampling & optimization |


- Main Page
- Related Pages
- Namespaces
- Classes
- Files

- File List
- File Members

All Classes Namespaces Files Functions Variables Typedefs Enumerations Enumerator Friends Macros Pages

- src
- stan
- agrad
- fwd
- prob
- univariate
- continuous

normal.hpp

Go to the documentation of this file.

1 #ifndef STAN\_\_AGRAD\_\_FWD\_\_PROB\_\_UNIVARIATE\_\_CONTINUOUS\_\_NORMAL\_HPP

2 #define STAN\_\_AGRAD\_\_FWD\_\_PROB\_\_UNIVARIATE\_\_CONTINUOUS\_\_NORMAL\_HPP

3

4 #include <boost/random/normal\_distribution.hpp>

5 #include <boost/random/variate\_generator.hpp>

6 #include <boost/utility/enable\_if.hpp>

7 #include <stan/agrad/partials\_vari.hpp>

8 #include <stan/math.hpp>

9 #include <stan/math/error\_handling.hpp>

10 #include <stan/meta/traits.hpp>

11 #include <stan/prob/constants.hpp>

12 #include <stan/prob/traits.hpp>

13

14 namespace stan {

15

16  namespace prob {

17

18  template <bool propto,

19  typename T\_y, typename T\_loc, typename T\_scale>

20  typename boost::enable\_if\_c<contains\_fvar<T\_y,T\_loc,T\_scale>::value,

21  typename return\_type<T\_y,T\_loc,T\_scale>::type>::type

22  normal\_log(const T\_y& y, const T\_loc& mu, const T\_scale& sigma) {

23  // static const char\* function = "stan::prob::normal\_log(%1%)";

24  // FIXME: add input checks

25

26  using std::log;

27  using stan::is\_constant\_struct;

28  using stan::math::value\_of;

29  using stan::prob::include\_summand;

30

31  // check if no variables are involved and prop-to

32  if (!include\_summand<propto,T\_y,T\_loc,T\_scale>::value)

33  return 0.0;

34

35  typename return\_type<T\_y,T\_loc,T\_scale>::type logp(0);

36

37  // log probability

38  if (include\_summand<propto>::value)

39  logp += NEG\_LOG\_SQRT\_TWO\_PI;

40

41  if (include\_summand<propto,T\_scale>::value)

42  logp -= log(sigma);

43

44  if (include\_summand<propto,T\_y,T\_loc,T\_scale>::value) {

45  typename return\_type<T\_y,T\_loc,T\_scale>::type z = (y - mu) / sigma;

46  logp -= 0.5 \* z \* z;

47  }

48

49  return logp;

50  }

51

52  }

53

54 }

55

56 #endif

traits.hpp

error\_handling.hpp

partials\_vari.hpp

constants.hpp

traits.hpp

stan::return\_type::type

boost::math::tools::promote\_args< typename scalar\_type< T1 >::type, typename scalar\_type< T2 >::type, typename scalar\_type< T3 >::type, typename scalar\_type< T4 >::type, typename scalar\_type< T5 >::type, typename scalar\_type< T6 >::type >::type type

**Definition:** traits.hpp:406

stan::is\_constant\_struct

Metaprogram to determine if a type has a base scalar type that can be assigned to type double...

**Definition:** traits.hpp:57

stan::math::value\_of

double value\_of(const T x)

Return the value of the specified scalar argument converted to a double value.

**Definition:** value\_of.hpp:24

stan::prob::include\_summand

Template metaprogram to calculate whether a summand needs to be included in a proportional (log) prob...

**Definition:** traits.hpp:35

math.hpp

stan::prob::normal\_log

boost::enable\_if\_c< contains\_fvar< T\_y, T\_loc, T\_scale >::value, typename return\_type< T\_y, T\_loc, T\_scale >::type >::type normal\_log(const T\_y &y, const T\_loc &mu, const T\_scale &sigma)

**Definition:** normal.hpp:22

stan::agrad::log

fvar< T > log(const fvar< T > &x)

**Definition:** log.hpp:15


---

[ Stan Home Page ]

*© 2011–2014,
Stan Development Team.*
